# Always Look on the Bright Side – Lessons Learned from Another Decade of Integrating Care

**DOI:** 10.5334/ijic.7513

**Published:** 2022-11-30

**Authors:** K. Viktoria Stein, Robin Miller, Edelweiss Aldasoro, Nicholas Goodwin

**Affiliations:** 1 Integrating Health and Care, Leiden University Medical Centre, NL; 2 Global Engagement for the College of Social Sciences, School of Public Policy. University of Birmingham, UK; 3 International Foundation for Integrated Care, Oxford, UK; 4 Central Coast Research Institute for Integrated Care, University of Newcastle and the Central Coast Local Health District, Level 10, 77A Holden Street, Gosford, NSW 2250, AU

**Keywords:** integrated care development, people-centred care, policy reforms, international development, social determinants of health

Another decade has gone by with considerable activity in policy, research and practice to develop, expand and scale integrated care around the world. Time to take stock again in this 20^th^ anniversary edition, which collates experiences from 21 countries through 13 policy and 1 research articles. Mostly written during the height of the pandemic, it also provides a vision of how to move forward, push boundaries and continue to bring people and services closer together in a time of crisis.

Our editorial reflecting on IJIC’s 10^th^ anniversary argued that integrated care had begun to move from a ‘*backwater activity and into mainstream thinking’* [1] and set out some likely emerging trends for the next decade including: the growth of e-health strategies; the move away from medicalised thinking towards embracing people as partners in care and promoting preventative activities; and system reforms in financing, governance and accountability that would lead to the growth in integrated care systems. But the authors also pointed to some prevailing shortcomings such as the imperative to build relationships and collaborate across all levels of the system and the need for more robust research and evaluation. The editorial concluded that integrated care was a ‘*principle whose time had come*’ with the hope that in ten years’ time integrated care would have become ‘*the norm rather than the exception in the way care is delivered in most countries*’.

Another ten years on and it would be fair to say these predictions are only partially true. Comparing the 21 countries, it is clear that integrated care as a public policy features in most countries. For example, various provinces of Canada have produced over 100 policy papers and strategies [2], while most other countries have been engaged in 1–3 national reforms promoting integrated care over the same period. In addition, international organisations including the European Union, the WHO and its regional offices have all produced frameworks and policies to strengthen integrated care, collaboration and people-centred services.

This is also reflected in the directionality of the reforms. Bengoa had famously called for the process of transformation towards integrated care to combine a top-down approach, creating the regulatory, financial and governance conditions for change, with a local and emergent bottom-up approach, allowing local teams of professionals and municipalities to implement the national framework according to local needs [3]. In 6 of the countries represented this advice has been followed. On the flip side this means however that in many countries top-down is still the prevailing way of implementation, with all its disadvantages and problems. And in no country is co-design with people and communities a key element in whatever direction.

Similarly, the focus has continued to be on disease management and on solutions to address the unsustainable rise in demand for [acute] care rather than health promotion and prevention. While calls have become louder to move towards more proactive management of health and wellbeing within communities, most integrated care models still focus on single chronic diseases or a specific target population and rarely consider social determinants of health. The notable exceptions being the USA, where Obamacare explicitly mentioned their importance [4], a few Canadian examples promoting co-design with the patients and communities [2] and Aotearoa New Zealand’s whanau philosophy, which has incorporated the Indigenous understanding of community wellbeing into public policies [5].

When it comes to collaboration on all levels of the system, again, the verdict is mixed. The focus continues to be on integration on the organisational level, vertically between secondary and primary care, and horizontally between health and social care providers. There are few examples, which break through these rather traditional boundaries.

Discussing the key messages and reflections from the past decade with participants and authors during a World Café at ICIC22, the collective agreement was that regions still don’t know “how to integrate”, and thus struggle with implementation. This is exacerbated by the vagueness of policies and a lack of measurable outcomes. When we talk about collective governance and leadership, the question arises of how to strike a balance between sharing accountabilities and staying autonomous. How much structure do we need to work together? The consensus seems to be that first and foremost, we need to be more flexible as individuals, organisations and systems. Top-down and bottom-up approaches need to inform each other through continuous learning cycles where policy frameworks allow innovative bottom-up approaches to trial the implications in practice and trigger adequate policy responses in turn. This would enable a more dynamic and community-driven evolution of integrated care. Somewhat confusingly, pilot projects remain a popular approach despite the frustrating fact that everyone agrees that they rarely seem to lead anywhere!

Looking back and looking forward, what are we left with? Many countries have sought to change their funding patterns to move away from fee-for-service models to create pooled budgets that may enable new approaches to care. Yet, our observation is that financial flows and incentives to support integrated care have not significantly progressed. While their importance of financial reform is acknowledged, few countries seem prepared to robustly embark on this daunting task. Likewise, digital health and information technologies have barely been allowed to unfold their full potential, often due to data protection laws, inaccessibility, or inexistence of data. Outcomes-based commissioning and continuous evaluation are still largely an academic promise rather than a reality, as outdated monitoring and performance measurement systems continue to be the easier way out. Constructive relationship building and interdisciplinary teamwork remain challenging as people often do not have the necessary time, energy or resources. The biggest challenge, however, remains the lack of person and community involvement, which sadly pervades all areas of integrated care.

So how far have we actually come in these past 10 years? To be fair, while the analysis may be frustrating for many, progress has still been made. There is a growing understanding of determinants of health and their importance, not least because the COVID-19 pandemic has put into stark relief what happens when they are ignored. Gradually, more and more policies and models are moving from disease management to population health management. Governance across sectors and systems remains difficult, but as the articles show, different approaches to accountability, financing and management across health and social care have been trialled. Just because they don’t always work out, does not mean nothing has been achieved. For example, the realisation that creating alliances and trust among stakeholders needs continuous investment is an important lesson which gradually finds its way into the plans and designs for implementation.

People and communities experiencing integrated care is still far from the norm, but the past decade has shown that with the increasing recognition of its importance and a wider understanding of the complexities, so the political interest and investment in integrated care solutions has multiplied. For the decade ahead the focus needs to shift to people and communities to give them permission, resources and the agency to come up with solutions tailored to their needs and to collaborate on an equal footing. Digital solutions must be implemented not as separate projects developed by specialists, but as an integral part of a wider implementation strategy. And another push needs to be made to include outcomes-based evaluation and monitoring as part of a wider data management strategy. We are on an exciting journey where integrated care is transitioning towards population-based solutions, and in the true tradition of emergent strategies, we cannot exactly say where it will lead us – but isn’t that what research and life are all about?
